# Editorial: Highlights in Physical Education and Pedagogy: 2021/22

**DOI:** 10.3389/fspor.2023.1202519

**Published:** 2023-05-12

**Authors:** Larissa Lara

**Affiliations:** Department of Physical Education, State University of Maringá, Maringá, Brazil

**Keywords:** risk and safety, skills, sports pedagogy, sedentary behavior, PE classes

**Editorial on the Research Topic** Highlights in Physical Education and Pedagogy: 2021/22

The call for the Frontiers in Sports and Active Living Research Topic *Highlights in Physical Education and Pedagogy: 2021/22* was put forth to be a selection of high-impact manuscripts produced by influential researchers on various topics related to the aforementioned areas of knowledge, with the potential to further reflection in a multidisciplinary forum. This joint effort by editors, authors, and the Frontiers team is animated by high expectations about how the selected themes would shine a light on the Research Topic itself and about the likely repercussion of these publications in the broader academic scenario. Once this stage of organizing the articles in the collection was concluded, some questions emerged: How would the published manuscripts on this Research Topic be received and appropriated by the broad forum of physical education and pedagogy researchers? Do these manuscripts have the potential to challenge and impact a multidisciplinary forum? What other topics would be highlighted in physical education and pedagogy in contemporary society, in addition to those that make up this collection, and which could motivate other Research Topics?

This Research Topic does not aim to be a thoroughly sufficient overview, given the diversity of approaches that integrate physical education and pedagogy in different contexts that have the potential to be highlighted. Therefore, these articles do not say everything! Nor do they intend to say so. They aspire for the incursion into peculiar themes that are not restricted to certain researchers, institutions, or countries. Such topics include the process of students' perception of learning life skills, sports pedagogy as an empirical-analytical academic discipline, risk and safety management practices in physical education classes, and determinants of physical activity and sedentary behavior in the school context.

The articles that make up this Research Topic are authored by researchers linked to higher education institutions in the United States, Norway, and Germany. Each manuscript brings investigative specificities that can be appropriated by different realities, given its potential for interlocution, cultural dialogues, and potential links to new findings. This peculiarity of the manuscripts encourages the establishment of connections and stimulates the production of knowledge in different local contexts.

The study developed by Jacobs et al. shows students' perceptions about learning life skills in the context of physical education classes. The authors use a teaching model of social responsibility (Teaching Personal and Social Responsibility—TPSR) to assess the perception that students have about the transposition of the knowledge acquired in the teaching environment into their daily lives. The results indicate that it is possible to give meaning to the knowledge acquired in class for the student's life and point to our social responsibility as educators. The research also points to the need to review our teaching methodologies so that the school's knowledge gains resonance in the student's life, in different spaces in which they build their identity.

The investigation of sports pedagogy and its position in the structure of modern sciences as an empirical-analytical academic discipline, carried out by Jaitner et al., problematizes the difference between claim and reality through the analysis of 212 scientific texts of sports-pedagogical provenance in the Germanic context. Based on Luhmann's systems theory, the authors analyze the mode, functions, and consequences of communication (disciplinary) in sports pedagogy, derived from conferences, collected editions, journals, and monographs, with emphasis on semantic forms and themes fixed in writing. The investigative findings lead to reflections on the field of sports pedagogy and notably transcend the investigated German reality.

The publication by Lise Porsanger and Leif Inge Magnussen explores risk and safety management (RSM) practices by teachers in physical education programs in Norway and reports a dearth of empirical studies on this topic. Through questionnaires and semi-structured interviews applied to teachers, the authors found the use of multiple strategies related to safety in physical education classes. For them, the physical education teacher can limit the exploration of movement by students, especially when teaching is permeated by fear of professional liability for the risk of accidents and physical injury to students. The theme is stimulating and challenges us to think about the fears and responsibilities that sometimes make it impossible for teachers to conduct teaching processes that explore creative and innovative practices in the school system. The manuscript leads us to think about gaps in teaching support in matters that involve risk in the teaching and learning process.

The article written by Jaitner et al. discusses the determinants of physical activity and sedentary behavior in physical education classes in German Elementary Schools, with the involvement of more than 300 students, in 11 schools. Data collection focused on physical activity concerning gender, grade, body mass index, active life, health behaviors, and the PE teachers' PE education status. The results are challenging as they indicate that physical activity levels and sedentary time are directly associated with gender, age, family habits, and PE teachers' PE education status, given the need for teacher didactic and methodological training to tackle these problems in the school system.

It should be noted that although this Research Topic foregrounds prominent themes in the fields of physical education and pedagogy, it could be expanded in terms of the number of themes and researchers from other institutions and countries, in addition to the North American and European outlines. In this direction, issues particular to different countries and continents could tense and provoke peculiar and enriching debates, expanding the scope of the Research Topic. I recognize, however, that unequal access to the publishing market also generates asymmetries in the production and dissemination of knowledge, often causing a lack of opportunities for researchers who could highlight new themes.

Finally, I emphasize that the topics presented in this Research Topic move us to examine our realities, to face issues that may be invisible in our investigative practices and need to be open to different interlocutions. The exercise of rationality that triggers alterity in the reading of these manuscripts makes it possible for us to be constantly crossed by the “other” (different from me), which reveals our fluid, dynamic identity constitution, for learning. This “doing in relation” awakens our social yearning, our affections, our condition as historical subjects, and our potential to learn “with”. I hope the highlights of this Research Topic can be read, appropriated, tensioned, and criticized. I hope also they contribute to the dynamics of the scientific field and to be shining a spotlight on other issues to provoke a fruitful debate. In that sense, what other highlights in physical education and pedagogy would you bring into the conversation?

